# Competency-based and problem-based learning methodologies: the WHO and ISS European Public Health Leadership Course

**DOI:** 10.1093/eurpub/ckae178

**Published:** 2025-03-25

**Authors:** Donatella Barbina, Joao Breda, Alfonso Mazzaccara, Alessandra Di Pucchio, Guglielmo Arzilli, Camilla Fasano, Christos Triantafyllou, Thanos Myloneros, Carlo Signorelli, Silvia Stacchini, Stefania Bocci, Lucia Dell’ Amura, Gaetano Pierpaolo Privitera, Luigi Bertinato, Natasha Azzopardi-Muscat, Silvio Brusaferro

**Affiliations:** Training Office, Italian National Institute of Health (Istituto Superiore di Sanità), Rome, Italy; WHO Office on Quality of Care and Patient Safety, World Health Organization Regional Office for Europe, Athens, Greece; Training Office, Italian National Institute of Health (Istituto Superiore di Sanità), Rome, Italy; Training Office, Italian National Institute of Health (Istituto Superiore di Sanità), Rome, Italy; Department of Translational Research and New Technologies in Medicine and Surgery, University of Pisa, Pisa, Italy; School of Medicine, University Vita-Salute San Raffaele, Milan, Italy; WHO Office on Quality of Care and Patient Safety, World Health Organization Regional Office for Europe, Athens, Greece; WHO Office on Quality of Care and Patient Safety, World Health Organization Regional Office for Europe, Athens, Greece; School of Medicine, University Vita-Salute San Raffaele, Milan, Italy; Training Office, Italian National Institute of Health (Istituto Superiore di Sanità), Rome, Italy; Training Office, Italian National Institute of Health (Istituto Superiore di Sanità), Rome, Italy; Division of Country Health Policies and Systems, World Health Organization Regional Office for Europe, Copenhagen, Denmark; Training Office, Italian National Institute of Health (Istituto Superiore di Sanità), Rome, Italy; Department of Translational Research and New Technologies in Medicine and Surgery, University of Pisa, Pisa, Italy; Training Office, Italian National Institute of Health (Istituto Superiore di Sanità), Rome, Italy; Division of Country Health Policies and Systems, World Health Organization Regional Office for Europe, Copenhagen, Denmark; Training Office, Italian National Institute of Health (Istituto Superiore di Sanità), Rome, Italy

## Abstract

The WHO European Programme of Work, 2020–2025 ‘United Action for Better Health in Europe’, backed up by the Regional Director’s vision, recognizes the need to invest in public health leadership towards addressing the multifaceted public health challenges in the WHO European Region. The WHO Regional Office for Europe in collaboration with the Italian National Institute of Health (Istituto Superiore di Sanità—ISS) developed the first European Public Health Leadership Course to support Member States in building their capacity. The course was delivered in blended modality over a period of 3 weeks (7–25 November 2022). It was structured according to the Competency-Based Education model and Problem-Based Learning methodology. Data analyses were conducted on the cohort of the course’s ‘Completers’. The formative assessment was conducted by a pre-post training questionnaire, while the summative assessment included three evaluation tools, in which participants were required to get a minimum overall mean score of 75/100 to pass the course. Thirty-eight participants passed all the summative tests out of 39 enrolled. The analysis of the multiple-choice questions showed an increase in knowledge. Survey results showed a high level of satisfaction. The course offered a mix of both theoretical and practical approaches, allowing participants to gain in-depth knowledge and develop skills that can be applied in their daily work. The successful completion of the course is expected to promote the development of the public health workforce in the eastern and southern parts of the WHO European Region.

## Introduction 

With the rising complexity and volume of public health challenges globally, public health professionals need to develop new competencies and act in partnership to tackle the main drivers of disease burden, address the determinants of health, and transform public health and health systems so that they put people at the centre.

In-depth and continuously updated training of public health professionals is essential. This enables them to support policy leaders and stakeholders with the most current scientific evidence. Such knowledge is crucial to prevent, protect against, mitigate, respond to, and recover from threats and hazards [1, 2]. This requires, among other aspects, reducing the fragmentation in the education of the public health workforce (PHW) among countries [3, 4]. Moreover, training represents a strategic element of which preparation and management cannot be addressed after the onset of the emergency, but it must be planned and made possible *a priori* [5]. The coronavirus disease 2019 (COVID-19) pandemic has underscored issues with public health system resilience and preparedness, emphasizing the need to strengthen their sustainability. It has also triggered a further reflection on new public health competencies. This presents an opportunity to review the public health competency framework recently launched by the World Health Organization (WHO) and the Association of Schools of Public Health in the European Region (ASPHER) through the lens of COVID‐19 [6].

The WHO European Programme of Work, 2020–2025 ‘United Action for Better Health in Europe’ clearly underscores the need to prepare the public health leaders of the future [7]. Using also lessons learned from the COVID-19 pandemic, the WHO Regional Office for Europe and the Italian Ministry of Health have worked together to identify opportunities to strengthen public health leadership, innovation, and governance—notably in the assisting of Member States in the southern and eastern parts of the WHO European Region. Within the framework of this joint endeavour, the WHO Regional Office for Europe and the Italian National Institute of Health (Istituto Superiore di Sanità—ISS) collaborated in developing a course specifically designed to train those embracing a career in public health with a focus on leadership, innovation, and governance.

ISS is the main centre for research, control, and technical-scientific advice on public health in Italy, working alongside the Ministry of Health, the Regions, and the National Health Service. Within the framework of the Italian Presidency of the G20 and in collaboration with the Ministry of Health, ISS, in line with the commitments made by the leaders of the G20 Countries at the Riyadh 2020 summit, launched in March 2021 an innovative training—Laboratorium, to strengthen the capacity and competencies of the PHW to better face the current challenge and to be ready to face possible future health challenges, through a One Health perspective [8, 9]. As stated in the Global Health Summit Rome Declaration [10] investing in the worldwide health and care workforce and developing mutually recognized competencies through education and training are key strategies. The Laboratorium initiative has enabled partnerships between G20 countries and other organizations, with WHO and its new Academy [11], in line with the need to renew the National Institutes of Public Health as Schools Without Walls [12], able to serve as nodes within networks for continuous health training.

In this context WHO and ISS designed the ‘European Public Health Leadership Course’ (PHLC). It was organized in a blended modality: Synchronous and asynchronous activities were delivered through the ISS’ e-Learning platform—EDUISS [13].

The PHLC incorporated public health insights with leadership competencies and experiences of public health leaders required in the field of public health. The faculty was composed of a variety of global pioneers and experts in public health and in correlated fields, including researchers, academics, policymakers, communication experts, and practitioners. The course was structured according to the Competency-Based Education (CBE) model and Problem-Based Learning (PBL) methodology.

In line with the model proposed by WHO [9, 14–16], CBE was incorporated as a methodology and educational approach to connect contents to the competencies required in the practice. To support the development of a competency-based public health leadership curriculum, the main reference was the framework proposed by Czabanowska *et al.* [17] on public health leadership competencies. This framework promotes a collaborative and shared leadership style, and it was built around a thematic framework consisting of 52 public health leadership competencies distributed among eight domains: Systems Thinking; Political Leadership; Building and Leading Interdisciplinary Teams; Leadership and Communication; Leading Change; Emotional Intelligence and Leadership in Team-based Organizations; Leadership Organizational Learning and Development; and Ethics and Professionalism [17].

PBL is a practice- and competence-based training methodology with a participant-led approach [18, 19]. Learning is realized in small groups of participants who analyse, study, discuss, and propose solutions to a problem inspired by work context, with a facilitator’s support. The methodology confronts the learner with the need to identify his own learning objectives to solve the problem and encourages the process of competence acquisition and the development of problem-solving skills. Since 2004, PBL has been progressively translated into the e-Learning context by the ISS training staff in three different models [20], based on the level of interaction between participants and facilitators: Low (high turnout, asynchronous, self-learning throughout the entire PBL cycle); intermediate (low number of participants, asynchronous, interaction with the facilitator on some PBL steps); high (small groups, synchronous, interaction with the facilitator on the entire PBL cycle, virtual classroom).

Assessing the efficiency of an educational program/course is crucial in ensuring its effectiveness and maximizing the educational outcomes for participants) [21].

The aim of this study was to describe the technical and methodological characteristics of the course and the main results in terms of participants’ characteristics and training impact.

## Methods

### Learning environment and methodology

The course was held in English and took place over 3 weeks (7–25 November 2022). The participants were expected to spend 53 h to complete the whole course, which was organized in a blended modality, including synchronous and asynchronous activities. All the course’s steps, from the enrolment to the final certification, were managed on the ISS e-Learning platform EDUISS, based on the Learning Management System (LMS) Totara 1.2. Its social constructivism approach [22], which emphasizes the social and collaborative nature of learning, its flexibility, and the availability of collaborative tools satisfy the aim of reproducing the PBL in the e-Learning context.

Based on the PBL high interaction model, participants were divided into six small groups composed of seven—eight participants and a facilitator to carry out group work. Two PBL cycles were provided, each of them consisting of the defined seven steps (Table 1):

**Table 1.** Structure of the blended course by weeks and learning objectives

Structure of the blended course		
Introductive resources	Course introduction; Informative e-communication resources (Forum Café; Participant Guide; Programme & Info; Faculty bios); Pre-training questionnaire.	
Course weeks		
*Course week*	*Learning objectives*	*Learning activities (and training modalities)*
Week 1 On-line (synchronous and asynchronous)	General objective: Define a common standard about leadership in public health Specific objectives: • Describe the role of the leader to implement effective solutions for public health. • Identify the core competencies of a leader in public health. • Identify the methods to make substantial and lasting changes in an organization to lead innovation.	• Lectures (Zoom sessions: pre-recorded and live) • Planning meeting—1 PBL cycle (Zoom session in separate rooms, small groups) • Monitoring meeting—1 PBL cycle (Forum and Zoom session in separate rooms, small groups) • Forum
Week 2 Residential	General objective: Discussing the main dimensions of leading oneself, others, and an organization. Specific objectives: • Identify the basic steps for building a working group. • Describe the basic steps for managing a working group. • Analyse the elements of effective communication in public health. • Identify the role of the leader in addressing ethical issues	• Solving meeting—1 PBL cycle (in presence, small groups) • Lectures and workshops (live sessions in presence; Zoom sessions: pre-recorded) • Planning meeting—2 PBL cycles (in presence, small groups) • Forum
Week 3 On-line (synchronous and asynchronous)	General objective: Identify multisectoral approaches to tackle public health challenges. Specific objectives: • Identify the attributes of the leader in public health emergency preparedness and response. • Identify fundamental components of system thinking approach. • Identify the main methods to improve collaborative leadership through interdisciplinary teams.	• Lectures (Zoom sessions: pre-recorded and live) • Monitoring Meeting—2 PBL cycles (Forum and Zoom session in separate rooms, small groups) • Solving meeting—2 PBL cycles (Zoom session in separate rooms, small groups) • Forum
Final resources and certification	Post-training questionnaire; Final certification test; Course quality evaluation questionnaire; Course activities satisfaction questionnaire (anonymous); Certificate of Attendance.

- Steps 1–5 (Clarify the concepts; Define the problem; Analyse the problem; Systematize the group’s hypotheses; Formulate learning objectives) lead to the *Planning meeting*, where participants analyse the problem, share previous knowledge, and identify their learning objectives to solve the problem;
- Step 6 (Study and individual work) conveys into the *Monitoring meeting*, which consists of researching, selecting, and studying learning materials to solve the problem, first individually and then within the group;
- Step 7 (Problem’s solution) conveys into the *Solution meeting*, where participants prepare an individual problem solution and then a group one, taking the most relevant parts of each individual proposal.

At the end of the two PBL cycles, each group elaborated a collective Problem solution from each individual one (see summative assessment). Also, the facilitator and the participants shared formative feedback on their group to improve the efficiency and effectiveness of the learning process.

The course was delivered in blended modality: The online weeks (1 and 3) were entirely delivered by EDUISS, which also hosted the materials and the forum of the residential activities (Week 2). Table 1 shows the structure of the blended course: Introductive resources, Week 1 online, Week 2 residential, Week 3 online, final resources, and certification. Each week consisted of specific learning activities and training modalities to achieve the identified learning objectives (Table 1). General and specific learning objectives were aligned to specific domains of the framework proposed by Czabanowska *et al.* [17] to promote a collaborative and shared leadership style. The learning objective assessment tools, namely the ‘Test Questions’, were also aligned to the same domains.

### Data collection and evaluation tools

The PHLC course was targeted to a maximum of 40 participants, according to the enrolment criteria defined by national health authorities and governments of Member States, mainly from the wider south-eastern WHO European Region and the Mediterranean Basin. 39 participants enrolled onto the course.

When registering on EDUISS, the following demographic and professional data about the participants were collected: Name, surname, gender, country, date of birth, and country of residence. Formative and summative assessment data were also collected.

Learner *formative assessment* was enhanced by self-assessment:

- *Pre-post training questionnaire:* Ten multiple-choice questions (MCQ), related to the course’s learning objectives and aligned to seven domains proposed by the WHO competency framework [17]: Systems thinking (two MCQ); Political leadership (one MCQ); Collaborative leadership: Building and leading teams (two MCQ); Leading change (one MCQ); Emotional intelligence and leadership in team-based organizations (two MCQ); Leadership, organizational learning and development (one MCQ); Ethics and professionalism (one MCQ). The MCQs were administered at the beginning (T0) and at the end of the course (T1). For each question, there were four possible answers only one of which was correct (0 points for wrong answer—1 point for correct answer). A minimal score was not required but completion was mandatory. At T1 participants received feedback, indicating wrong answers.

*The summative assessment included three evaluation tools.* To pass the course, participants were required to get a minimum overall mean score of 75/100 out of the three summative tests:

- *Two Individual Problem Solution* tools were scheduled at the end of the first and the second PBL cycles. Four assessment criteria were used to evaluate the individual problem solution (coherence with the learning objectives; completeness; relevance; clarity) on a 0–100 point scale. The total score was calculated as a mean of the evaluation of each criterion, where higher scores reflected higher compliance with the criteria. Additional feedback was also provided to participants.
- *The final certification test (MCQs)* consisted of a set of 20 questions related to the course learning objectives. For each question there were four possible answers; only one of them was correct (0 points for wrong answer—1 point for correct answer on a 100-point scale).

At the end of the course, participants were also required to fill in the mandatory satisfaction questionnaires:

- *Course quality evaluation questionnaire:* Twenty-two closed questions about Learning Methodology, Contents, and e-Learning platform functioning. All the questions were evaluated on a 5-point Linkert scale (1 = do not agree at all, 2 = do not agree; 3 = neither agree nor disagree; 4 = agree; 5 = very much agree), where higher values indicate higher levels of satisfaction. Also, two open questions were provided to collect opinions on ‘Positive aspects of the course’ and ‘Suggestions to improve the quality of the course’.
- *Course activities satisfaction questionnaire:* An anonymous questionnaire of 40 closed questions was used to measure participants’ satisfaction with lectures, experiential learning activities, social activities, and organizational and logistical aspects. The first 32 questions (1.1.1–1.3.7) were evaluated on a 1- to 10-point Likert scale, with ‘1’ being ‘minimum interest’ and ‘10’ being ‘maximum interest’. The rest of the questions (2.1–4.1) were evaluated on a 5-point Linkert scale. One open question was also provided to collect opinions on the organizational aspects of the residential week.

Participants who successfully completed all learning activities received a Certificate of Attendance.

### Statistical analysis

Data analyses were conducted on the cohort of ‘Completers’, including participants who completed the course: (1) filled out the two self-assessment tests (T0 pre-test, T1 post-test), (2) passed three assessment tests with a mean score of at least of 75%, and (3) filled out the satisfaction course and activity questionnaires. ‘Non-completers’ were participants who enrolled in the course but did not complete it for voluntary withdrawal or who did not start it at all and participants who failed in passing the assessment tests (not passed).

Quantitative variables were tested for normality by the Shapiro–Wilk test. Counts and percentages were calculated for the qualitative variables (QVs); means and standard deviations (SD) or medians and interquartile ranges (IQR; Q1–Q3) were calculated for QVs that, respectively, follow or didn’t follow a normal distribution.

Differences between each person’s pre-test (T0) and post-test (T1) scores were calculated (one-group pre-post study design), assuming that values at T0 were lower than T1. Comparisons between pre- and post-test were performed using a Wilcoxon test for two related samples because the differences between participants’ total scores on the 10 MCQs before and after training did not follow a normal distribution. Moreover, mean, median, and SD are presented for participants’ final certification test scores. The results of course and activities satisfaction questionnaires were analysed and presented as mean and SD for each item.

A nominal significance level of *P* ≤ 0.05 was set for all statistical analyses. Analyses were performed using the statistical software Statistical Package for Social Sciences (SPSS) version 28.0 (IBM Corp., Armonk, NY).

## Results

### Course participants

The 39 enrolled participants came from 20 countries of the southern and eastern WHO European Region: Albania, Armenia, Azerbaijan, Bosnia and Herzegovina, Bulgaria, Croatia, Georgia, Greece, Hungary, Italy, Malta, Montenegro, North Macedonia, Republic of Moldova, Romania, Serbia, Slovenia, Spain, Türkiye, as well as Kosovo. (All references to Kosovo in this document should be understood to be in the context of the United Nations Security Council resolution 1244 (1999).) The 17 Italian participants came from 15 different regions (out of 21 Italian Regions).

Thirty-eight out of the 39 enrolled participants passed all the summative tests (completers).

Most of the completers (23 out of 38; 60.5%) were males. The completer’s mean age was 39.2 years (SD 7.1; age range 24–56).

### Formative pre- and post-test results

The median overall pre- and post-training scores were 4 (3, 6) and 8.5 (5, 10), respectively. The number of correct and wrong answers for each of the 10 MCQs before and after training shows that at the end of the course, the number of correct answers increased in 9 of 10 questions (90%). Statistically significant differences in mean frequency rankings were observed (*z* = 4.15 and *P* < 0.001). Considering the seven domains linked to the 10 questions, the major improvements were associated with System Thinking (Q8 Diff. +22) and Emotional intelligence and leadership in team-based organizations (Q4 Diff. +20); the minor improvements were associated with Collaborative leadership: Building and leading interdisciplinary teams (Q1 Diff. 0) and Ethics and professionalism (Q3 Diff. +2) (Table 2).

**Table 2.** Number of correct answers to each of the 10 multiple-choice questions (MCQs) relating to the course’s learning objectives before (T0) and after training (T1). *N* = 38

Domain^a^	Competency^a^	MCQs	Correct answers before the course (T0) *N* (%)	Correct answers after the course (T1) *N* (%)	Diff.
Collaborative leadership: building and leading interdisciplinary teams	17. Model effective group process behaviours including listening, dialoguing, negotiating, rewarding, encouraging, and motivating	Question 1. Which of the following answers shows the correct sequence of the main steps needed to build a working group:	28 (73.7)	28 (73.7)	0
Leading change	31. Make strategic decisions based on recognized values, priorities, and resources	Question 2. Which of the following activities can be relevant to make substantial and lasting changes in an organization, particularly in order to lead innovation:	25 (65.8)	37 (97.4)	+12
Ethics and professionalism	49. Make a clear declaration of any conflict of interest that is likely to affect your leadership or decision-making and take appropriate action to minimize this	Question 3. Considering the role of the leader, which of the following can be an example of addressing an ethical issue:	17 (44.4)	19 (50)	+2
Emotional intelligence and leadership in team-based organizations	38. Respond appropriately to the positive criticism of others about your own behaviour or performance	Question 4. Which of the following competences is relevant to strengthen emotional intelligence in team-based organizations:	12 (31.6)	32 (84.2)	+20
Leadership, organizational learning, and development	42. Advocate for learning opportunities within the organization	Question 5. Which of the following is the correct definition of the role of the leader according to Stanley Kaplan:	24 (63.2)	27 (71.1)	+3
Collaborative leadership: building and leading interdisciplinary teams	17. Model effective group process behaviours including listening, dialoguing, negotiating, rewarding, encouraging, and motivating	Question 6. Which of the following answers shows the correct sequence of the main steps needed to manage a working group:	11 (28.9)	25 (65.8)	+14
Emotional intelligence and leadership in team-based organizations	34. Demonstrate awareness of the impact of your own beliefs, values, and behaviours on your own decision-making and the reactions of others	Question 7. The terms ‘determinants of vaccination choice’ refer to which of the following factors?	27 (71.1)	35 (92.1)	+8
Systems thinking	2. Synthesize and integrate divergent viewpoints for the good of an organization	Question 8. Based on the recent literature (>2010), which of the following are leadership-related domains?	3 (7.9)	25 (65.8)	+22
Political leadership	10. Evaluate and determine appropriate actions regarding critical political issues	Question 9. Which of the following can be a key competence to tackle an emergency?	7 (18.4)	24 (63.2)	+17
Systems thinking	2. Synthesize and integrate divergent viewpoints for the good of an organization	Question 10. Which of the following competences is relevant to strengthen a system thinking approach:	11 (28.9)	28 (73.7)	+17

a Czabanowska *et al.* [17].

### Summative assessment

Descriptive statistics are presented in Supplementary Table S1 for the three evaluation tools administered. Overall, the average score was 8.92 points (±0.59), with a minimum of 7.54 and a maximum of 9.92. The scores for each evaluation tool were as follows: For the first Individual Problem solution (1), the mean was 8.45 (±0.94; range 5–10), while for the second Individual Problem solution (2), the mean was 9.32 (±0.43; range 8.25–10). For the summative MCQs, the mean value of correct answers was 8.99 (±1.18; range 5–10).

### Course quality evaluation questionnaire results

A summary of the overall distribution of answers to each question, the mean and SD, as well as the total score for each question, appears in Supplementary Table S2.

**Figure 1.**
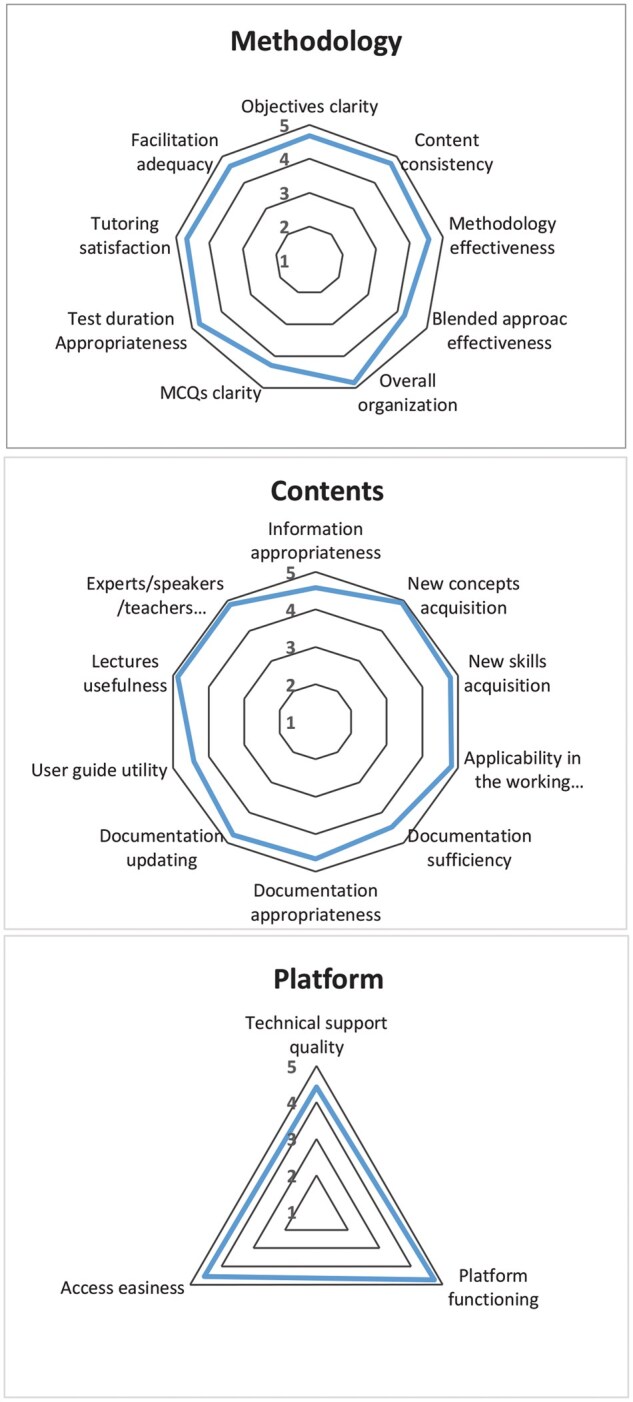
Course quality evaluation questionnaire results on Learning Methodology, Contents, and e-Learning platform functioning.

Participants reported scores >4 for the three components (Fig. 1). They were very satisfied with the learning methodology, the adequacy of the contents, and the functioning of the e-Learning platform.

### Course activities satisfaction questionnaire results

In 28 (87.5%) lectures, participants rated their interest level at 9 or 10, indicating that most lectures were very interesting (Questions 1.1.1–1.3.7). In response to the question, ‘Were the social activities proposed during the residential week of the course interesting?’ 35 out of 38 (92.1%) participants answered, ‘all or almost all’, while 81.6% (31) of the participants stated that they were very satisfied with the organizational and logistical aspects of the residential part of the course. Supplementary Table S3 shows the overall distribution of responses, the total score, and the mean and SD for each question.

## Discussion

Public health plays an essential role in society to prevent disease, prolonging life expectancy, and promoting community health. Moreover, the pandemic has highlighted the need for a globally coordinated response to invest in prevention, preparedness, and response to address upcoming health emergencies [23].

This study aimed to analyse the results of the European PHLC, carried out to train future public health leaders coming from different countries of the southern and eastern WHO European Region. Our goal was to create a training path based on proven effective learning methods [24, 25], which we identified in the PBL and CBE models.

A blended modality, consisting of a combination of residential and online learning, was adopted. For some authors blended modality is considered as effective and satisfying as in-person modality [25–29]. Moreover, it is considered a good way to facilitate interaction and robustly reproduce the PBL cycle on the entire learning path [25].

The high interaction PBL model was chosen for the online component, considering that leads to higher levels of satisfaction and better results in knowledge increasing in comparison to the low interaction modality [24, 30]. In the high interaction model, the PBL steps are closer to the residential modality due to the active and synchronous interaction between participants and facilitators, increasing motivation and physiological arousal in participants [31, 32].

The learning experience has been enhanced with the residential component, working face-to-face in small groups, as in the original PBL model. Participants expressed an overall appreciation for the course’s methodological approach. In the Course quality evaluation questionnaire, the ‘methodology’ category had an average of 4.6 (out of 5), and 84% of participants expressed a high or very high appreciation for the blended modality.

Particular attention was given to the formative and summative assessments. The formative assessment is an essential moment of the learning process, and it helps participants to personally track their learning achievements about the topic via the pre–post questionnaire. This is confirmed by the results of the pre-course and post-course tests, which show a significant increase in correct answers. This result, as reported in the literature on the relationship between scores and appreciation/validity of a training path [33–35] also confirms the quality of the course.

Furthermore, some variability is always observed in the questions’ answers, due to the participants’ background and expectations [28]. For example, on Pre-Post Test Question 1 (Table 2), associated with the ‘Collaborative leadership: Building and leading interdisciplinary teams’ domain, there is no improvement in scores from T0 to T1, highlighting hypothetically that participants with a public health background already had a good knowledge on that area. Otherwise, there was a significant improvement in scores on the questions associated with the domains ‘Systems thinking’ and ‘Emotional intelligence and leadership in team-based organizations’, showing a lack of starting knowledge on these fields. All the domains were covered by Lectures and Experiential learning activities (Supplementary Table S3), allowing participants to achieve any starting knowledge gaps.

Although starting from different backgrounds and knowledge levels, the PBL method enabled all the participants to achieve good knowledge results [36] on the different domains. The facilitator’s feedback also helps participants to improve their group-work skills, which are fundamental for leadership.

A short MCQ summative test was also added since part of the assessment was directed to the levels of ‘understanding’ and ‘remembering’ [37], The test mean score was very good. However, as some authors point out, MCQs are not fully appropriate to assess the competencies acquired through the PBL approach, as it should be based on performance and not only upon giving correct answers [38]. The high interaction model allowed the setting of summative assessments that match effectively the procedural approach of a PBL session as the evaluation of the individual Problem solution. For this reason, the course incorporated an evaluation system consistent with the PBL methodology. This included both an objective assessment framework based on MCQs, focusing on knowledge as well as two Individual Solutions where participants applied the PBL method, as detailed in Table 1. Both individual solutions produced positive results, with the second yielding particularly good results—an improvement often observed with repeated application of PBL cycles. Individual Solution 2 shows an improvement in average scores compared to Individual Solution 1, reflecting the participants’ progressive and expected familiarization with PBL proper assessment tools.

Having a specific competence framework for leadership [17] has made it possible to identify learning objectives in line with certain domains and then select the topics to be covered. During the 3 weeks of the course three general objectives were addressed: Define a common standard about leadership in public health; discuss the main dimensions of leading oneself, others, and an organization; identify multisectoral approaches to tackle Public Health challenges. The objectives were achieved through various learning activities and lectures carried out by a set of leading global public health speakers.

The specific framework was chosen because it encourages a collaborative and shared leadership style and embodies specific public health leadership qualities like the capacity to identify and involve stakeholders in cross-disciplinary projects to enhance public health; to make sure that organizational practices are in line with changes in the public health system and the larger social, political, and economic environment; and the capacity to form coalitions, alliances, and partnerships to improve community health.

No significant critical issues were detected, as the WHO and ISS staff were in constant contact with the participants. Small adjustments of settings were quickly made in progress. We modified the access settings to the resources, providing more time for the completion of the same to facilitate the course’s completion.

This experience represents an important basis for future improvements and experimentations with the support of the National Institutes of Public Health to develop networks and Schools without walls in which also leadership skills can be conveyed and reinforced. Thanks to the online weeks, this course reached participants coming from 20 different countries. The participants, all professionals in the Public Health sector, despite the strict timetable planned and their job commitment, had the possibility to attend the activities all day long.

Blended learning could be considered as a possible way to facilitate interaction and to robustly reproduce the PBL cycle on the platform, nonetheless, it requires high involvement of an adequate staff endowed with different skills (technology, planning, communication, web writing, training methodology). Virtual classroom, crucial for a good interaction on the PBL’s steps, consisting of interactions in small groups (6–8 max for each facilitator), requires greater commitment from participants and facilitators.

It should also be considered that the implementation of a learning project requires considerable planning and particular attention to the preparation of learning materials, which must be adequate to PBL and technology requirements (format, size, copyright, etc).

Finally, the course offered a mix of both theoretical and practical approaches, allowing participants to gain in-depth knowledge and develop skills that can be applied in their daily work. The successful completion of the course is expected to promote the development of the PHW in the eastern and southern parts of the WHO European Region. All participants in the course were encouraged to share the knowledge and best practices they have acquired with their peers, which should be of great use in their current and future positions, enabling them to be better prepared to tackle the public health challenges of tomorrow.

Furthermore, by encouraging international cooperation, the policies will become more adaptable and responsive to health workforce crises [39]. The course can be considered as a pilot experience, promoting the creation of regional and international networks on leadership. Other leadership training initiatives are currently underway in several other European countries and regions. Once a network of leadership initiatives has been created, it will be possible to better respond to public health emergencies, analysing the long-term training impact.

## Supplementary Material

ckae178_Supplementary_Data

## Disclaimer

The authors affiliated with the World Health Organization (WHO) are alone responsible for the views expressed in this publication and they do not necessarily represent the decisions or policies of the WHO.
Key points

- WHO Regional Office for Europe and the Italian National Institute of Health (ISS—Istituto Superiore di Sanità, Rome, Italy) organized the European Public Health Leadership Course to train those embracing a career in public health focusing on leadership, innovation, and governance.
- The course was structured over 3 weeks (online and residential) and based on the Competency-Based Education (CBE) model and Problem-Based Learning (PBL) methodologies.
- The course offered theoretical and practical approaches, allowing participants to gain in-depth knowledge and develop skills that can be applied in their daily work. The successful completion of the course is expected to promote the development of the public health workforce in the eastern and southern parts of the WHO European Region.
- Quality training of public health experts is essential to support policy leaders and stakeholders to prevent, protect against, mitigate, respond to, and recover from threats and hazards.
- National Institutes of Public Health as Schools without walls could support testing and implementation of training models to also strengthen leadership competencies.
