# Supplementary material for: Competency-based and problem-based learning methodologies: the WHO and ISS European Public Health Leadership Course
Source: Eur J Public Health. 2025 Mar 25;35(Suppl 2):ii21–8. doi: 10.1093/eurpub/ckae178 (PMC11933797; doi:10.1093/eurpub/ckae178)
Supplement: ckae178_Supplementary_Data [file ckae178_supplementary_data.docx]

# **Supplementary Material**

**Supplementary Table 1.** Descriptive statistics for the three tests included in the summative evaluation (N=38)

|  | **N** | **Min** | **Max** | **Median** | **IQR** | **Mean** | **SD** |
| --- | --- | --- | --- | --- | --- | --- | --- |
| **Individual Problem solution (1)** | 38 | 5 | 10 | 8,50 | 8, 9 | 8.45 | 0.94 |
| **Individual Problem solution (2)** | 38 | 8.25 | 10 | 9,25 | 9, 9.75 | 9.32 | 0.43 |
| **Summative McQs** | 38 | 5 | 10 | 9,25 | 8, 10 | 8.99 | 1.18 |
| **Total score** | **38** | **7.54** | **9.92** | **8.92** | **8.56, 9.42** | **8.92** | **0.59** |

*Note.* SD, Standard deviation; Min, Minimum value; Max, Maximum value; IQR; interquartile range (Q_1_-Q_3_)

**Supplementary Table S2.** Overall distribution of responses to each question (Course quality evaluation questionnaire)

| **Questions- Learning Methodology** | **Responses** | | | | | **Total score; Mean ± SD** |
| --- | --- | --- | --- | --- | --- | --- |
|  | **1-Strongly disagree** | **2-Disagree** | **3-Neither agree nor disagree** | **4-Agree** | **5-Strongly agree** |  |
| **1.1 The course objectives were clear** |  |  |  | 12 (31.6%) | 26 (68.4%) | **178; 4.68 ± 0.47** |
| **1.2 The content was consistent with the course objectives** |  |  |  | 10 (28.3%) | 28 (73.7 %) | **180; 4.73 ± 0.45** |
| **1.3 The educational method was effective** |  |  | 2 (5.3%) | 12 (31.6%) | 24 (63.2%) | **174; 4.58 ± 0.60** |
| **1.4 The blended learning approach (online and residential) was effective** |  |  | 6 (15.8%) | 17 (44.7%) | 15 (39.5%) | **161; 4.24 ± 0.71** |
| **2.4 The overall organisation of the course (course structure, timing, evaluations) was satisfactory** |  |  |  | 6 (15.8%) | 32 (84.2%) | **184; 4.84 ± 0.37** |
| **2.6 The questions of the tests were sufficiently clear** |  |  | 5 (13.2%) | 17 (44.7%) | 16 (42.1%) | **163; 4.29 ± 0.69** |
| **2.7 The amount of time available to complete the test was adequate** |  |  |  | 10 (28.3%) | 28 (73.7 %) | **180; 4.74 ± 0.45** |
| **3.2 The quality of the guidance for this distance learning course was satisfactory** |  |  | 1 (2.6%) | 10 (28.3%) | 27 (71.1%) | **178; 4.68 ± 0.47** |
| **3.4 The quality of the facilitation was adequate** |  |  | 2 (5.3%) | 10 (28.3%) | 26 (68.4%) | **176; 4.63± 0.59** |
| **Questions-Contents** | **Responses** | | | | | **Total score; Mean ± SD** |
|  | **1-Strongly disagree** | **2-Disagree** | **3-Neither agree nor disagree** | **4-Agree** | **5-Strongly agree** |  |
| **1.5 The amount and quality of information were appropriate for my knowledge** |  |  | 2 (5.3%) | 12 (31.6%) | 24 (63.2%) | **174; 4.58 ± 0.60** |
| **1.6 I have learnt new concepts** |  |  |  | 3 (7.9%) | 35 (92.1%) | **187; 4.92 ± 0.27** |
| **1.7 I have acquired new skills** |  |  | 1 (2.6%) | 6 (15.8%) | 31 (81.6%) | **182; 4.79 ± 0.47** |
| **1.8 I can apply what I have learnt in this course to my professional context** |  |  | 1 (2.6%) | 5 (13.2%) | 32 (84.2%) | **183; 4.82 ± 0.45** |
| **2.1 The provided documents were sufficient to acquire the necessary knowledge** |  |  | 1 (2.6%) | 18 (47.4%) | 19 (0.5%) | **170; 4.47 ± 0.56** |
| **2.2 The provided documents were of appropriate quality** |  |  |  | 13 (34.2 %) | 25 (63.2%) | **177; 4.66 ± 0.48** |
| **2.3 The provided documents were up to date with the most recent literature** |  |  | 1 (2.6%) | 8 (21.1%) | 29 (76.3%) | **180; 4.74 ± 0.50** |
| **2.5 The user guide was helpful to understand the learning path** |  |  | 3 (7.9%) | 16 (42.1%) | 19 (0.5%) | **168; 4.42 ± 0.64** |
| **2.8 The lectures were useful for my work and/or personal development** |  |  |  | 5 (13.2%) | 33 (86.8%) | **185; 4.87± 0.34** |
| **3.3 The experts/speakers/teachers were competent and prepared** |  |  |  | 5 (13.2%) | 33 (86.8%) | **185; 4.87± 0.34** |
| **Questions- E-learning platform functioning** | **Responses** | | | | | **Total score; Mean ± SD** |
|  | **1-Strongly disagree** | **2-Disagree** | **3-Neither agree nor disagree** | **4-Agree** | **5-Strongly agree** |  |
| **3.1 The quality of the technical support for this distance learning course was satisfactory** |  |  | 2 (5.3%) | 8 (21.1%) | 28 (73.7%) | **168; 4.42 ± 0.64** |
| **4.1 The online learning platform functioned properly** |  |  | 1 (2.6%) | 8 (21.1%) | 29 (76.3%) | **180; 4.74 ± 0.50** |
| **4.2 Platform access modes were simple and immediate** |  |  | 2 (5.3%) | 13 (34.2 %) | 23 (60.5%) | **173; 4.55 ± 0.60** |

*Note.* SD, Standard deviation

**Supplementary Table S3.** Overall distribution of responses to each question (Course activities satisfaction questionnaire)

| **Questions - 1. Lectures** | **Response (N, %)** | | | | | | | | | | | | | | | | | | | **Total score; Mean ±SD** |  |
| --- | --- | --- | --- | --- | --- | --- | --- | --- | --- | --- | --- | --- | --- | --- | --- | --- | --- | --- | --- | --- | --- |
|  | 1* | 2 | 3 | | 4 | | 5 | | | 6 | 7 | | | 8 | 9 | | | 10** | |  |  |
| **1.1.1 Future of Public Health** |  |  |  | |  | 1  (2.6%) | | | 1  (2.6%) | | | 5  (13.2%) | | 7  (18.4%) | | 16  (42.1%) | | | 8  (21%) | **326; 8.57 ± 1.19** |  |
| **1.1.2 Seven attributes for public health leaders** |  |  |  | |  | |  | | |  | 2  (5.3%) | | | 7  (18.4%) | 12  (31.6%) | | | 17  (44.7%) | | **348; 9.16 ± 0.92** |  |
| **1.1.3 The global epidemics of non-communicable diseases** |  |  |  | |  | |  | | | 2  (5.3%) | 4  (10.5%) | | | 7  (18.4%) | 12 (31.5%) | | | 13  (34.2%) | | **334; 8.79 ± 1.19** |  |
| **1.1.4 The innovation perspective in public health** |  |  |  | |  | |  | | | 1  (2.6%) | 5  (13.2%) | | | 9  (23.7%) | 14  (36.8%) | | | 9  (23.7%) | | **329; 8.66 ± 1.07** |  |
| **1.1.5 Digital transformation and digital leadership** |  |  |  | |  | |  | | | 2  (5.3%) | 6  (15.8%) | | | 8  (21%) | 13  (34.2%) | | | 9  (23.7%) | | **325; 8.55 ± 1.18** |  |
| **1.1.6 Develop innovative and technological strategies to resolve the global healthcare burden of NCDs** |  |  |  | |  | |  | | | 2  (5.3%) | 6  (15.8%) | | | 7  (18.4%) | 11  (28.9%) | | | 12  (31.5%) | | **329; 8.66 ± 1.07** |  |
| **1.1.7 Tackling health inequities through public health policy** |  |  |  | |  | |  | | | 1  (2.6%) | 3  (7.9%) | | | 9  (23.7%) | 14  (36.8%) | | | 11  (28.9%) | | **336; 8.82 ± 1.04** |  |
| **1.1.8 Improving health systems: the people’s voice survey** |  |  |  | |  | |  | | | 1  (2.6%) | 6  (15.8%) | | | 9  (23.7%) | 11  (28.9%) | | | 11  (28.9%) | | **329; 8.66 ± 1.07** |  |
| **1.1.9 Chasing the rainbow: social determinants of health in the post-pandemic** |  |  |  | |  | |  | | | 1  (2.6%) | 3  (7.9%) | | | 6  (15.8%) | 17  (44.7%) | | | 11  (28.9%) | | **338; 8.89 ± 1.01** |  |
| **1.1.10 Big data: how can data help us counter the world’s biggest challenges?** |  |  |  | |  | |  | | | 1  (2.6%) | 4  (10.5%) | | | 9  (23.7%) | 12  (31.5%) | | | 12  (31.5%) | | **334; 8.79 ± 1.09** |  |
| **1.2.1 The core competencies for public health leaders** |  |  |  | |  | |  | | | 4  (10.5%) | 6  (15.8%) | | | 6  (15.8%) | 12  (31.5%) | | | 10  (26.3%) | | **322; 8.42 ± 1.39** |  |
| **1.2.2 Leadership for effective and sustainable precision health** |  |  |  | |  | | 1  (2.6%) | | | 3  (7.9%) | 6  (15.8%) | | | 7  (18.4%) | 12  (31.5%) | | | 9  (23.7%) | | **319; 8.34 ± 1.41** |  |
| **1.2.3 Mindful Leadership in the Healthcare sector** |  |  |  | |  | |  | | |  | 2  (5.3%) | | | 4  (10.5%) | 11  (28.9%) | | | 21  (55.3%) | | **355; 9.34 ± 0.88** |  |
| **1.2.4 How to get public health communication right** |  |  |  | |  | |  | | | 3  (7.9%) |  | | | 3  (7.9%) | 12  (31.5%) | | | 20  (52.6%) | | **350; 9.21± 1.14** |  |
| **1.2.5 Leadership development training** |  |  |  | |  | |  | | |  | 2  (5.3%) | | | 2  (5.3%) | 8  (21%) | | | 26  (88.4%) | | **362; 9.52± 0.83** |  |
| **1.2.6 Social Media and Effective Leadership** |  |  |  | | 3  (7.9%) | |  | | | 5  (13.2%) | 5  (13.2%) | | | 5  (13.2%) | 9  (23.7%) | | | 11  (28.9%) | | **296; 8.1± 1.84** |  |
| **1.2.7 Leadership development training** |  |  |  | |  | |  | | | 1  (2.6%) | 1  (2.6%) | | | 3  (7.9%) | 8  (21%) | | | 25  (65.8%) | | **359; 9.44± 0.95** |  |
| **1.2.8 Public health policy making, politics and evidence** |  |  |  | |  | |  | | | 2  (5.3%) | 3  (7.9%) | | | 10  (26.3%) | 10  (26.3%) | | | 13  (34.2%) | | **333; 8.76± 1.17** |  |
| **1.2.9 Leading High Performance Teams during complex situations** |  |  |  | |  | |  | | |  | 3  (7.9%) | | | 3  (7.9%) | 13  (34.2%) | | | 19  (50.0%) | | **352; 9.26 ± 0.92** |  |
| **1.2.10 Building resilient leadership for public health** |  |  |  | | 1  (2.6%) | |  | | | 3  (7.9%) | 6  (15.8%) | | | 5  (13.2%) | 12  (31.5%) | | | 11  (28.9%) | | **322; 8.42 ± 1.46** |  |
| **1.2.11 Leadership and Communication: the challenge of future public health** |  |  |  | | 1  (2.6%) | |  | | | 4  (10.5%) | 5  (13.2%) | | | 10  (26.3%) | 7  (18.4%) | | | 11  (28.9%) | | **316; 8.26 ± 1.48** |  |
| **1.2.12 How to tackle infodemics and health misinformation** |  |  |  | | 1  (2.6%) | | 1  (2.6%) | | | 2  (5.3%) | 1  (2.6%) | | | 6  (15.8%) | 9  (23.7%) | | | 18  (47.4%) | | **345; 9.0 ± 1.55** |  |
| **1.2.13 How to lead policy making to tackle health inequality** |  |  |  | |  | |  | | | 1  (2.6%) | 3  (7.9%) | | | 10  (26.3%) | 15  (39.5%) | | | 9  (23.7%) | | **332; 8.89 ± 1.03** |  |
| **1.2.14 Effective Communication: Well-crafted visuals** |  |  |  | |  | |  | | |  |  | | | 5  (13.2%) | 5  (13.2%) | | | 28  (73.7%) | | **365; 9.60 ± 0.71** |  |
| **1.2.15 Public Health Leadership: effective negotiation and conflict management** |  |  |  | |  | |  | | |  | 3  (7.9%) | | | 5  (13.2%) | 17  (44.7%) | | | 13  (34.2%) | | **344; 9.05± 0.90** |  |
| **1.3.1 The social aspects of vaccine hesitancy, uncertainty and scepticism** |  |  |  | |  | |  | | |  | 4  (10.5%) | | | 8  (21%) | 15  (39.4%) | | | 11  (28.9%) | | **337; 8.87 ± 0.96** |  |
| **1.3.2 Digital leadership for public health emergency and response** |  |  |  | |  | |  | | | 1  (2.6%) | 3  (7.9%) | | | 10  (26.3%) | 10  (26.3%) | | | 9  (23.7%) | | **332; 8.89 ± 1.03** |  |
| **1.3.3 Understanding mental health as a key public health opportunity** |  |  |  | |  | |  | | |  | 5  (13.2%) | | | 8  (21%) | 14  (36.8%) | | | 11  (28.9%) | | **335; 8.82 ± 1.00** |  |
| **1.3.4 Broad societal engagement for health sustainability** |  |  |  | |  | |  | | |  | 5  (13.2%) | | | 10  (26.3%) | 15  (39.5%) | | | 8  (21%) | | **330; 8.68 ± 0.96** |  |
| **1.3.5 Climate change: An important target for early intervention in mental health** |  |  |  | |  | |  | | | 1  (2.6%) | 5  (13.2%) | | | 7  (18.4%) | 13  (34.2%) | | | 12  (31.5%) | | **334; 8.79 ± 1.19** |  |
| **1.3.6 From Alma-Ata to Astana: Bridging the gap** |  |  |  | |  | |  | | |  | 7  (18.4%) | | | 7  (18.4%) | 13  (34.2%) | | | 11  (28.9%) | | **332; 8.89 ± 1.03** |  |
| **1.3.7 Leading yourself in emergency response** |  |  |  | |  | | 2  (5.3%) | | |  | 3  (7.9%) | | | 3  (7.9%) | 10  (26.3%) | | | 20  (52.6%) | | **345; 9.0 ± 1.55** |  |
| **Questions - 2. Experiential learning activities** | **Response (N, %)** | | | | | | | | | | | | | | | | | | | **Total score; Mean ±SD** |  |
|  | **1-Strongly disagree** | | | **2-Disagree** | | | | **3-Neither agree nor disagree** | | | | | **4-Agree** | | | | **5-Strongly agree** | | |  |  |
| **2.1 I think the mindfulness sessions will be useful in my life and/or work** |  | | | 2  (5.0%) | | | | 6  (15.8%) | | | | | 13  (34.2%) | | | | 17  (44.7%) | | | **159; 4.18 ± 0.90** |  |
| **2.2 I think the workshops in communication will be useful in my life and/or work** |  | | |  | | | |  | | | | | 7  (18.4%) | | | | 31  (81.6%) | | | **271; 4.82 ± 0.39** |  |
| **2.3 I think the workshops in leading myself will be useful in my life and/or work** |  | | |  | | | | 1  (2.6%) | | | | | 11  (28.9%) | | | | 26  (68.4%) | | | **177; 4.66 ± 0.53** |  |
| **2.4 I think the workshops in leading others and a manage a group will be useful in my life and/or work** |  | | |  | | | |  | | | | | 11    (28.9%) | | | | 27  (71.1%) | | | **179; 4.7± 0.46** |  |
| **2.5 I think the insights we received on social media including misinformation will be useful in my life and/or work** |  | | |  | | | | 4  (10.5%) | | | | | 12  (31.6%) | | | | 22  (57.9%) | | | **170; 4.47 ± 0.69** |  |
| **2.6 I think the workshops in effective negotiation and conflict management will be useful in my life and/or work** |  | | |  | | | | 1  (2.6%) | | | | | 16  (42.1%) | | | | 21  (55.3%) | | | **172; 4.52 ± 0.56** |  |
| **Questions - 3. Social activities** | **Response (N, %)** | | | | | | | | | | | | | | | | | | |  |  |
|  | **1- I did not participate in social activities** | | | **2- Only a few or none** | | | | **3-Less than half** | | | | | **4-More than half** | | | | **5- All or almost all** | | | **Total score; Mean ±SD** |  |
| **3.1 Were the social activities proposed during the residential week of the course interesting?** |  | | | 1  (2.6%) | | | |  | | | | | 2  (5.3%) | | | | 35  (92.1%) | | | **185; 4.86 ± 0.53** |  |
| **Questions - 4. Organizational and logistical aspects** | **Response (N, %)** | | | | | | | | | | | | | | | | | | | **Total score; Mean ±SD** |  |
|  | **1- Very dissatisfied** | | | **2-Dissatisfied** | | | | **3-Neither satisfied nor dissatisfied** | | | | | **4-Satisfied** | | | | **5- Very satisfied** | | |  |  |
| **4.1 How satisfied are you with the organizational and logistical aspects of the residential part of the course?** | 1  (2.6%) | | |  | | | |  | | | | | 6  (15.8%) | | | | 31  (81.6%) | | | **180; 4.74 ± 0.72** |  |

*:1, minimum interest

**:10, maximum interest

*Note.* SD, Standard deviation
